# Improvement of Manganese Feroxyhyte’s Surface Charge with Exchangeable Ca Ions to Maximize Cd and Pb Uptake from Water

**DOI:** 10.3390/ma13071762

**Published:** 2020-04-09

**Authors:** Evgenios Kokkinos, Chasan Chousein, Konstantinos Simeonidis, Sandra Coles, Anastasios Zouboulis, Manassis Mitrakas

**Affiliations:** 1 Department of Chemistry, Aristotle University of Thessaloniki, 54124 Thessaloniki, Greece; evgenios@chem.auth.gr (E.K.); zoubouli@chem.auth.gr (A.Z.); 2 Department of Chemical Engineering, Aristotle University of Thessaloniki, 54124 Thessaloniki, Greece; cheng_auth_2013@hotmail.com (C.C.); sandycoles@hotmail.com (S.C.); 3 Ecoresources P.C., 15-17 Giannitson-Santaroza Str., 54627 Thessaloniki, Greece; simeonidis@ecoresources.gr

**Keywords:** Cadmium, lead, adsorption, ion exchange, manganese feroxyhyte, surface modified adsorbent

## Abstract

The surface configuration of tetravalent manganese feroxyhyte (TMFx) was appropriately modified to achieve higher negative surface charge density and, hence, to improve its efficiency for the removal of dissolved Cd and Pb mostly cationic species from water at pH values commonly found in surface or ground waters. This was succeeded by the favorable engagement of Ca^2+^ cations onto the surface of a mixed Mn-Fe oxy-hydroxide adsorbent during the preparation step, imitating an ion-exchange mechanism between H^+^ and Ca^2+^; therefore, the number of available negatively-charged adsorption sites was increased. Particularly, the calcium coverage can increase the deprotonated surface oxygen atoms, which can act as adsorption centers, as well as maintain them during the subsequent drying procedure. The developed Ca-modified adsorbent (denoted as TMFx-Ca) showed around 10% increase of negative surface charge density, reaching 2.0 mmol [H^+^]/g and enabling higher adsorption capacities for both Cd and Pb aquatic species, as was proved also by carrying out specific rapid small-scale column tests, and it complied with the corresponding strict drinking water regulation limits. The adsorption capacity values were found 6.8 μg·Cd/mg and 35.0 μg·Pb/mg, when the restructured TMFx-Ca adsorbent was used, i.e., higher than those recorded for the unmodified material.

## 1. Introduction

Currently, worldwide interest regarding the quality issues of drinking water is intense, as the respective sources are becoming increasingly scarce. The continuous industrial development of the past decades has led to the enforcement of stricter legislation, regarding the emissions and concentrations of pollutants in the environment. Among the listed high priority pollutants [1], Cd and Pb are regulated by very low Drinking Water Regulation Limits (DWRLs), being 5 and 10 μg/L, respectively [2].

According to the relevant literature, the most promising available technology, able to reduce the dissolved concentrations of Cd and Pb below their respective DWRLs, is considered to be adsorption [3,4]. The coagulation–precipitation process is also commonly implemented for the removal of heavy metals [5,6]; however, the disadvantages of this process, such as the production and management of relatively high chemical sludge quantities and the relevant higher labor cost, usually restrict its application only for the larger scale treatment units, or when treating highly polluted industrial wastewaters. In contrast, the adsorption process presents significant advantages, including the preservation of important raw water quality characteristics and the lower capital and operating costs, especially when applied to purify natural waters, containing rather low initial metal concentrations, i.e., in the scale of μg/L.

The choice of an appropriate adsorbent type for the specific needs of water treatment is crucial. The major categories of examined adsorbents for the removal of heavy metals can be mainly categorized into: organic-based [7,8], inorganic [9], agricultural by-products [10], and ion-exchange resins [11]. Unfortunately, most studies have evaluated the efficiency of adsorbents by calculating the maximum adsorption capacity values (Q_max_), which can only be obtained when applying considerably high initial concentrations of the examined pollutants (usually in the order of several mg/L). It is worth noting that this approach practically results in most cases to residual concentrations that are significantly exceeding the respective DWRLs [4]. Consequently, the majority of relevant publications overlook the need to evaluate the efficiency of adsorbents by succeeding residual concentrations (C_e_) efficiently below the current DWRLs, and to estimate their removal capacity at C_e_ = DWRL (denoted as Q_DWRL_ value) within a natural water matrix [7,8,9,10,11,12,13].

The surface charge density is a critical parameter that determines the efficiency of an adsorbent to remove ionic species of toxic metals from a water matrix. For instance, adsorbents with excess negative surface charge density are expected to favor the capture of positively charged hydrated metal cations, such as Cd and Pb [14]. In a previous study, the tetravalent manganese feroxyhyte material (TMFx), which is a Mn(IV) partially substituted iron oxy-hydroxide, was applied as a negatively charged adsorbent, able to achieve high Q_DWRL_ values, when examined for the removal of Cd, Ni, and Hg toxic metals from aqueous solutions [3]. Furthermore, it was shown that the surface charge distribution depends on the protonation (positive charge)–deprotonation (negative charge) rate of TMFx’s oxygen atoms content. The aim of this study was to modify appropriately the surface of TMFx material, aiming to increase the negative surface charge density (due to the deprotonated oxygen atoms) and, thus, to improve the adsorption efficiency of specific heavy metal cations.

The specific methodology to improve the surface charge density by the attachment of specific ions onto an adsorbent during its synthesis (preparation) step was previously demonstrated by our research group. Specifically, SO_4_^2−^ ions were used to increase the positive surface charge density of iron oxy-hydroxides (FeOOH), resulting in the enhanced adsorption/removal of arsenic. It was proved that the SO_4_^2−^ anions were adsorbed onto the surface of FeOOH during synthesis, favoring the elevation of positive surface charge density and, consequently, the adsorption capacity for the removal of H_2_AsO_4_^−^/HAsO_4_^2−^ anionic species, mainly through the implementation of an anion exchange mechanism [15,16]. In the present study, the synthesis route was performed on the basis of Ca cation adsorption excess in order to maximize the negative surface charge density, to effectively maintain the active adsorption sites during the final drying process, and, hence, to improve the adsorption capacity for Cd and Pb toxic metals.

## 2. Materials and Methods

### 2.1. Synthesis of Adsorbents

Calcium-modified tetravalent manganese feroxyhyte (TMFx-Ca) was synthesized by the co-precipitation/oxidation of FeSO_4_·H_2_O (40 g/L, 10 L/h) with the addition of KMnO_4_ (25 g/L, 3 ± 0.3 L/h) and CaCl_2_·2H_2_O (52 g/L, 1 L/h) solutions, to maintain the Ca concentration at the 1 ± 0.1 g Ca^2+^/L level. For comparison reasons, the corresponding tetravalent manganese feroxyhyte (TMFx) was prepared according to the procedure optimized by Kokkinos et al. [14], but without the presence of Ca salt. The reagents were fed into a two-stage continuous stirred-tank reactor (CSTR) (BIOTEX S.A., Thessaloniki, Greece), operating with residence times of 1 h for each reactor (Figure S1). The synthesis pH was adjusted to 9 ± 0.1 by the drop-wise addition of NaOH solution (30 wt.%), while the redox potential was adjusted to 600 ± 50 mV, by regulating the KMnO_4_ solution flow rate. The suspension, collected from the reactor outflow, was initially thickened for 24 h, washed several times with distilled water to lower the conductivity of the liquid phase below 2 mS/cm, and it was subsequently subjected to solid–liquid separation by centrifugation and finally dried for 4 h at 90° C. The received dried adsorbent was ground and sieved in the form of fine powder (with lower than 63 μm diameter) and used for the batch adsorption experiments, whereas the larger size granules (0.25–0.50 mm) were used for the rapid small-scale column tests.

### 2.2. Characterization

The dominant structural phase of the studied adsorbents was determined by X-ray diffractometry (XRD) (Rigaku, Tokyo, Japan) using a water-cooled Rigaku UltimaPlus diffractometer with CuKa radiation, a step size of 0.05° and a step time of 3 s, operating at 40 kV and 30 mA. The specific surface area was estimated by applying the Brunauer–Emmett–Teller (BET) model [17], performing nitrogen gas adsorption measurements, by using a micropore surface area analyzer (Self-construction, Thessaloniki, Greece) at liquid N_2_ temperature (77 K). Field Emission-Scanning Electron Microscopy (FE-SEM) images were obtained by using a Quanta 200 ESEM FEG FEI microscope (Quanta, Berkhamsted, UK) with a field-emission gun, operating at 15–30 kV. This system was equipped also with an EDAX energy-dispersive X-ray spectroscopy analyzer that recorded the distribution of elements in the structure of examined samples.

The measurement of main surface charge properties was performed by determining the isoelectric point (IEP), the point of zero charge (PZC), and the (negative) charge density of each adsorbent, noting that the IEP determination requires the plotting of the corresponding zeta-potential curve. For this purpose, 50 mg/L suspension of finely powdered adsorbent was dispersed in an electrolyte solution (0.01 M NaNO_3_) and this was equilibrated at various pH values (3–10), by adding either HNO_3_ or NaOH dilute solutions (see Figure S2). The electrophoretic velocity of charged particles was determined by a Rank Brothers Micro-electrophoresis Apparatus Mk II (Rank Brothers, Cambridge, UK). PZC was determined by the potentiometric mass titration method, in which the suspensions of examined adsorbents were equilibrated in various pH values (i.e., in the range 4–10) [18]. The differences between the initial and the equilibrium pH (ΔpH) were plotted as a function of the adjusted pH value, with the intersection point of the curve in the x-axis to indicate the respective PZC value. Fine powdered adsorbent (10 g/L) was dispersed in 0.001, 0.01, and 0.1 M NaNO_3_ solutions and equilibrated to pH 11. These dispersions were titrated by 0.1 M HNO_3_ solution until the pH value 3 was reached (see Figure S3). The PZC was found as the point of intersection for the three ionic strengths in the plot of surface charge density, which was defined by the difference of acid volume required to achieve the same pH value in the measured sample and in the relevant blank titration. The reverse process was followed to determine the negative charge density. Specifically, the dispersions were adjusted to the pH value 3, and then they were titrated by adding 0.1 M NaOH solution until the known PZC was reached.

Iron, manganese, calcium, and sodium content in the examined adsorbents were determined by Flame Atomic Absorption Spectrophotometry (FAAS), using a Perkin Elmer AAnalyst 800 instrument (Perkin Elmer, Waltham, MA, USA), after the dissolution of solid samples in 6 N HCl. The specific manganese valence was also calculated by titration (see Text S1).

### 2.3. Adsorption Experiments

Standard stock solutions of Cd and Pb (1000 mg/L) were prepared according to Standard Methods [19]. The working solutions were prepared by the complete dilution of stock solutions in nature-like water, complying with the National Sanitation Foundation (NSF) protocols. To prepare the NSF water, 252.0 mg NaHCO_3_, 12.14 mg NaNO_3_, 0.178 mg NaH_2_PO_4_·H_2_O, 2.21 mg NaF, 70.6 mg NaSiO_3_·5H_2_O, 147.0 mg CaCl_2_·2H_2_O, and 128.3 mg MgSO_4_·7H_2_O were diluted in 1 L of distilled water.

The adsorption capacity was evaluated by performing batch experiments, as well as Rapid Small-Scale Column Tests (RSSCT), under experimental conditions that simulate a full-scale treatment process. The batch experiments were carried out in 300 mL conical flasks by dispersing 10–40 mg of fine powdered adsorbents (<63 μm) into 200 mL of pollutant (toxic metal) solution in NSF water matrix and adjusting the pH value within the range 6–8. After shaking the flasks for 24 h, the suspension was filtered through 0.45 μm pore size membrane filter and the initial and residual concentrations of metals were determined by Graphite Furnace—Atomic Absorption Spectrophotometry (GF-AAS), using a Perkin Elmer AAnalyst 800 instrument.

For the RSSCT experiments, a glass column with an internal diameter of 1.1 cm and height of 40 cm, fitted with PTFE valves, caps, and a glass frit at the base, was used. The column was filled with 10 g of TMF or TMFx-Ca granules (of size 0.25–0.50 mm and bulk density 0.45 g/mL) to obtain bed height 23 cm and fed from the top by a dosing pump with 200 μg/L concentration of toxic metals’ NSF aqueous solution at pH 7.2 ± 0.1. By setting the hydraulic loading of 350 ± 20 mL/h, the Empty Bed Contact Time (EBCT) was calculated as 3.8 ± 0.2 min. Water samples were periodically collected from the effluent and analyzed to determine the respective residual metal concentrations.

### 2.4. Leaching Behavior

The leaching of adsorbed Cd and Pb from the spent (consumed) adsorbents was performed after the end of RSSCT experiments; it was estimated through the appropriate leaching tests, performed according to the standard leaching test EN 12457 protocol [20]. The obtained results indicate their compliance to the current environmental regulations, regarding the environmentally safe disposal of used adsorbents and considering the stabilization of removed metals’ content.

## 3. Results and Discussion

### 3.1. Structural and Physicochemical Characterization

**Figure 1:**
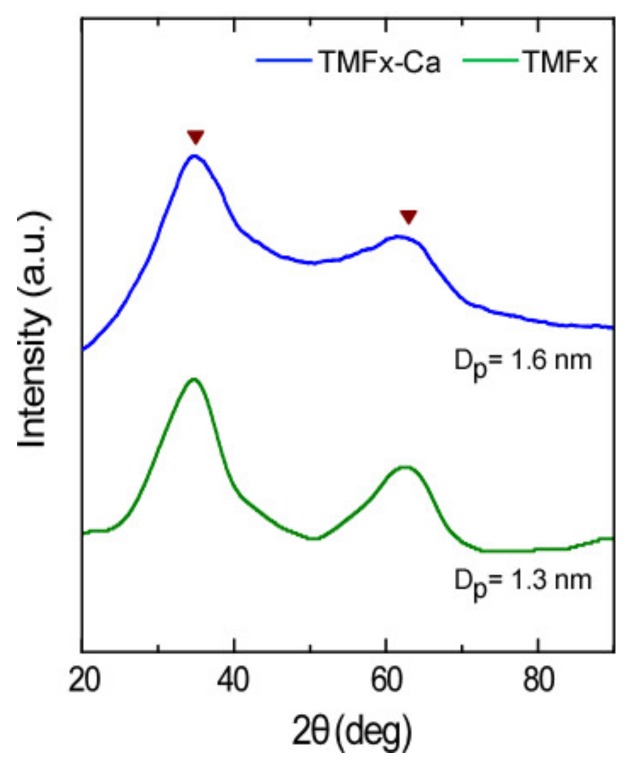
XRD diagrams of TMFx and TMFx-Ca adsorbents. Triangles indicate the Mn-feroxyhyte diffraction angles, according to the ICDD/JCPDS database PDF#14-0557 [21]. The mean crystal size (D_p_) values correspond to the calculated particle dimensions.

The high oxidative conditions, occurring during the preparation procedure of TMFx and TMFx-Ca materials, as determined by the high redox potential values (600 ± 50 mV), favor a rather low crystallinity order [14] of these adsorbents that can be verified by the observed broadening of diffraction peaks in the XRD diagrams (Figure 1). The main crystalline phase for both examined adsorbents, i.e., with and without the presence of Ca, was identified as Mn-feroxyhyte. Therefore, the presence of calcium during the synthesis of TMFx-Ca material did not modify the crystalline structure. In contrast, the mean crystal size (D_p_ = 1.6 nm) of TMFx-Ca, as estimated by applying the Scherrer’s equation in the main peaks, was found to be slightly higher than that of the TMFx (D_p_ = 1.3 nm).

**Figure 2:**
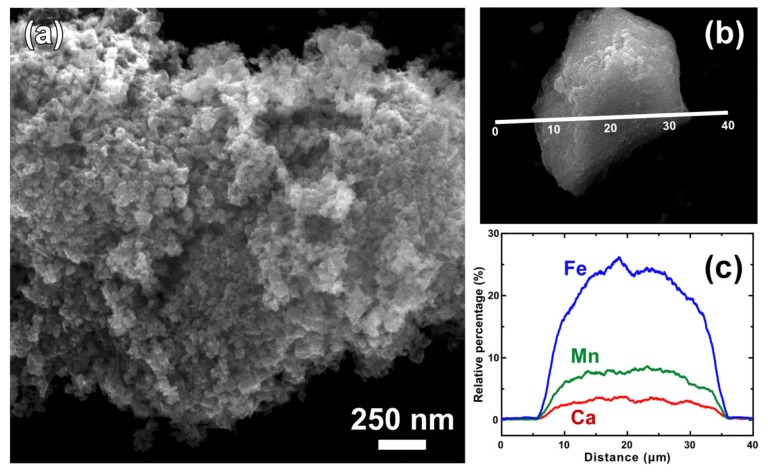
(**a**) Higher magnification FE-SEM image showing the nanoscale morphology of TMFx-Ca; (**b**) lower magnification FE-SEM image of one granule from this adsorbent; and (**c**) the respective elemental distribution of Fe, Mn, and Ca, as obtained by line-scan analysis in this granule.

Figure 2a,b shows the surface morphology of TMFx-Ca at different magnifications. At the lower magnification, this adsorbent appears to be comprised of massive grains; however, at the higher magnification, it reveals a sponge-like structure, formed by the aggregation of primary particles with dimensions below 10 nm, in agreement with the evaluation of crystal size (XRD diagrams).

The indicative elemental analysis, performed in an expanded region of one granule, showed the homogeneous ordering of Fe and Mn at the atomic level (Figure 2c), thus verifying the isomorphic substitution of Fe by Mn within the feroxyhyte structure and a constant mass ratio of around 3:1. In addition, the presence of adsorbed Ca with an average Ca/(Fe + Mn) ratio of around 0.07 was also determined. The EDAX results were further verified by the physicochemical characterization of both adsorbents (Table 1). The content of Fe and Mn remained almost constant in both adsorbents, whereas, in the Ca-modified adsorbent (TMFx-Ca), the significant replacement of Na by Ca was also observed. These results verify that due to the presence of Ca^2+^ during the oxy-hydroxide’s synthesis, Na was almost quantitatively substituted by Ca at several adsorption sites. Conclusively, the higher selectivity of TMFx material to Ca adsorption rather than Na resulted in the formation of modified TMFx-Ca adsorbent.

**Figure 3:**
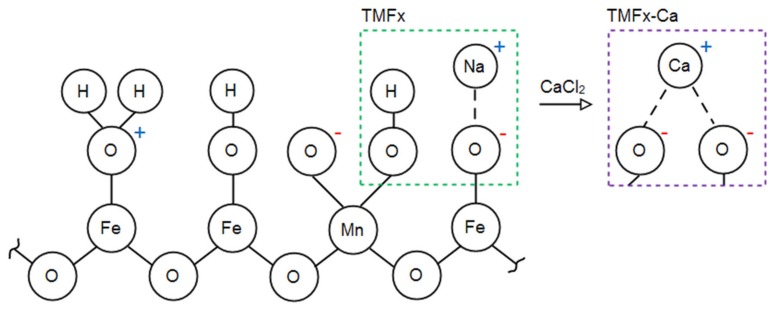
Proposed surface charge distribution (positive, negative, and neutral) for the TMFx and TMFx-Ca adsorbents.

Regarding the surface properties of adsorbents, the specific surface area of TMFx-Ca material was found to be lower than that of the TMFx. According to the literature, this can be attributed to the adsorption of calcium cations that may increase the Face/Edge sharing ratio of the created Fe/Mn(O,OH)_6_ chains [22]. In contrast, the TMFx-Ca material exhibited an increased surface charge density, possibly related to the presence of adsorbed calcium, which inhibits the loss of active sites during the subsequent drying step of the solid (after synthesis). The gradual exchange of Ca^2+^ by the Cd^2+^ or Pb^2+^ cations during the adsorption process contribute to the observed improved adsorption capacity. The protection of active sites by the adsorbed calcium may also explain the lower IEP value (3.6) recorded for the case of TMFx-Ca, when compared to the corresponding value for TMFx (5.9, Figure S2), where the number of deprotonated active sites [–O^−^] was found to decrease (Figure 3). The relevant effect of (other) adsorbed ions, such as SO_4_^2−^, on the IEP values and the resulting surface charge density of iron oxy-hydroxides, was also previously reported [15]. On the other hand, the PZC values show only slight variations between the two adsorbents, due to the presence of different surface-adsorbed cations, i.e., Na^+^ and Ca^2+^ (see also Figure S3).

### 3.2. Modification of Surface Charge

It has been shown that the surface charge density of TMFx adsorbent is related mainly to the protonation and deprotonation of oxygen atoms, without excluding also the case of neutral sites. During the preparation step under the applied alkaline conditions, the excess of OH^−^ presence would favor the deprotonation of oxy-hydroxide surfaces, increasing the negative surface charge density [14]. On the other hand, during the preparation under acidic conditions, the excess of H^+^ presence can lead to an increased positive surface charge density [23]. Therefore, the pH conditions during the preparation of these adsorbents is expected to highly influence the specific type and density of TMFx surface charge (Figure S4). Furthermore, the contribution of other ions, which might be also present in the reactor during the synthesis procedure, cannot be considered as negligible. It is well-known that, by applying exchangeable ions, such as Na^+^, Ca^2+^, and SO_4_^2-^, during the preparation step of adsorbents, the surface charge volume and distribution may be modified [14,24], and it can also be stabilized/maintained during the following drying process, as aforementioned. In fact, the surface H^+^ can be exchanged by other cations during the synthesis step, i.e., by Na^+^ in the TMFx case and by Ca^2+^ in the TMFx-Ca material [25]. The proposed surface complexation of examined materials (Figure 3) indicates that Na^+^ is weakly bound with one oxygen atom in the case of TMFx, while Ca^2+^ is attached to two atoms in the TMFx-Ca material, as dictated by the valence state of each exchangeable cation. In addition, the substitution of cations in the case of TMFx-Ca can be attributed to the lower affinity of Na^+^, as compared with Ca^2+^ [26]. As a result, the TMFx-Ca presented a slight increase of measured negative surface charge density from 1.8 to 2.0 mmol H^+^/g (Table 1), which, however, has shown significantly higher adsorption capacities, regarding the removal of the examined toxic metal cations (i.e., Cd and Pb).

### 3.3. Cadmium and Lead Speciation

**Figure 4:**
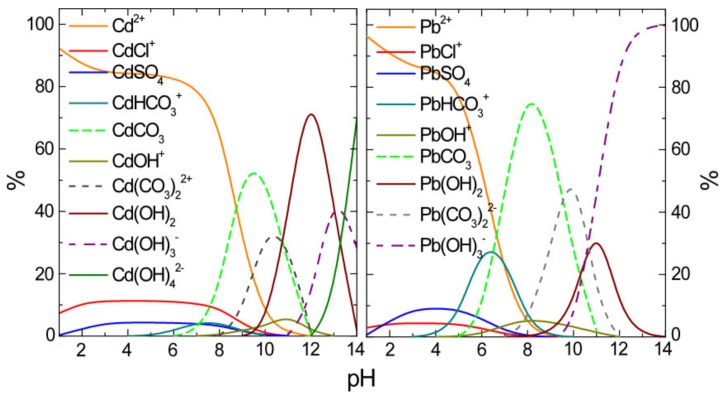
Speciation diagrams for the examined metals (Pb and Cd) at the initial concentration of 0.1 mg/L, according to the MINTEQ version 3.0 software in NSF water matrix.

Apart from the adsorbent’s properties, the adsorption efficiency is also highly dependent on the adsorbate’s speciation. For this purpose, the speciation diagrams of Cd and Pb in NSF water matrix were designed, by using the Visual MINTEQ version 3.0 software (Figure 4). The composition of NSF water and the concentration of each of the examined pollutants were used as input for the respective program calculations.

Since the aim of this study was the treatment of natural waters to become drinking water, the equilibrium pH values of interest were focused within the range of 7–8. At this pH range, the dominant specie of lead was PbCO_3_ due to its low solubility in water (K_sp_ = 1.5 × 10^−13^). Other species of this metal found at significant concentrations were the dissolved ones Pb^2+^ and PbHCO_3_^+^ [27]. The dominant species of cadmium were found to be Cd^2+^ and CdCl^−^, while the CdCO_3_ (K_sp_ = 5.2 × 10^−12^) can be present at significant concentration levels, but only at pH values higher than 7.5. Due to the low solubility of carbonate salts and, to obtain reliable results, specific attention should be given to avoid precipitation, when applying low initial metal concentrations; otherwise, the relative error of these procedures may increase substantially. Both examined metals have been found to exhibit mainly positively charged ions (Figure 4), thus requiring a specialized adsorbent that possesses a correspondingly high negative charge to assist their adsorptive removal.

### 3.4. Adsorption Isotherms

#### 3.4.1. Cd Adsorption

**Figure 5:**
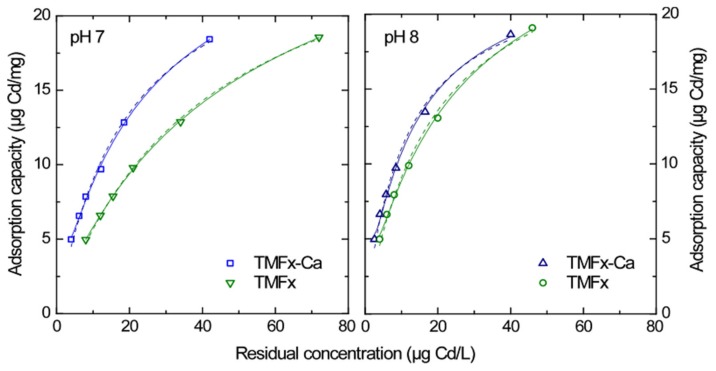
Cd adsorption isotherms in NSF water matrix and for pH values 7 and 8. Solid and dashed lines represent the Freundlich and Langmuir fittings, respectively.

Since the TMFx and TMFx-Ca materials were synthesized to treat contaminated water for potable use, the adsorption isotherms were conducted by applying the natural-like NSF water matrix and considering residual concentrations close to and below the respective DWRL for Cd and representative pH values 7 and 8. Figure 5 presents the corresponding data for Cd fitting, according to the Freundlich [Q_e_ = K_F_·C_e_^1/n^] and Langmuir [Q_e_ = Q_max_·K_L_·C_e_/(1 + K_L_·C_e_)] main adsorption equations, where Q_e_ represents the amount of adsorbed metals per mass of adsorbent at the equilibrium concentration C_e_, Q_max_ is the maximum adsorption capacity, *K_F_* and *n* are Freundlich constants related to adsorption capacity and intensity, and *K_L_* is the Langmuir constant. The good fitting of adsorption data to both models can be explained by the low adsorption load, relating with the low equilibrium concentration range, which indicates a more or less monolayer coverage [3]. The main adsorption parameters of both adsorbents, regarding Cd removal, are presented in Table 2. These data reveal that, at pH 7, TMFx-Ca shows higher adsorption capacity at DWRL (Q_5_ = 5.88 μg Cd/mg) than TMFx (Q_5_ = 3.94 μg Cd/mg), due to the higher negative surface charge density of TMFx-Ca material.

An increase of adsorption efficiency was observed at the pH value 8 for both adsorbents with Q_5_ values 7.29 μg Cd/mg for TMFx-Ca and 5.91 μg Cd/mg for TMFx. This increase was attributed to the enhanced negative surface charge density, due to the higher pH value since the IEP values of adsorbents were 3.6 and 5.9, respectively (Table 1). Furthermore, the variations in the affinity constants of the applied models (i.e., the *n* value for Freundlich and the *K_L_* for Langmuir) were proportional to the adsorption efficiency. However, it must be clarified that the Q_max_ values, resulting from the Langmuir equation, can be considered as indicative only, as they were calculated by the extrapolation of low equilibrium concentrations. These experimental data focus on achieving low residual concentrations (as imposed by the current legislation) in order to get more realistic evaluation of adsorption capacity for the cases of Cd (Q_5_) and Pb (Q_10_) and to predict the efficiency of this procedure in larger scale water treatment plants.

#### 3.4.2. Pb Adsorption

**Figure 6:**
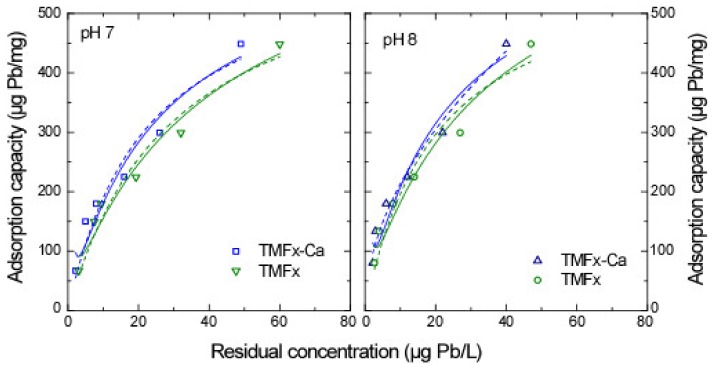
Pb adsorption isotherms in NSF water matrix for pH values 7 and 8. Solid and dashed lines represent the Freundlich and Langmuir fittings, respectively.

Considering the Pb adsorption isotherms, when low initial concentrations were applied (i.e., ≤1 mg Pb/L), both TMFx-Ca and TMFx adsorbents produced residual concentrations below 20 μg Pb/L, which limited the fitting to Freundlich and Langmuir models. However, for higher initial metal concentrations (i.e., 1–10 mg Pb/L), the adsorption isotherms (Figure 6) showed substantially higher adsorption capacities (Table 3), which were considered as rather abnormal. On further investigation, the filtration of standard solutions with concentrations higher than 1 mg Pb/L through a 0.2 μm pore size membrane filter revealed a white precipitate (Figure S5), consisting of Pb(OH)_2_·PbCO_3_. For this reason, the initial concentration as applied in the following RSSCT experiments was selected to be 200 μg Pb/L. These experimental data demonstrate that several published studies on Pb adsorption within a natural water matrix may require further verification, due to possible precipitation occurrences.

### 3.5. Column Tests

**Figure 7:**
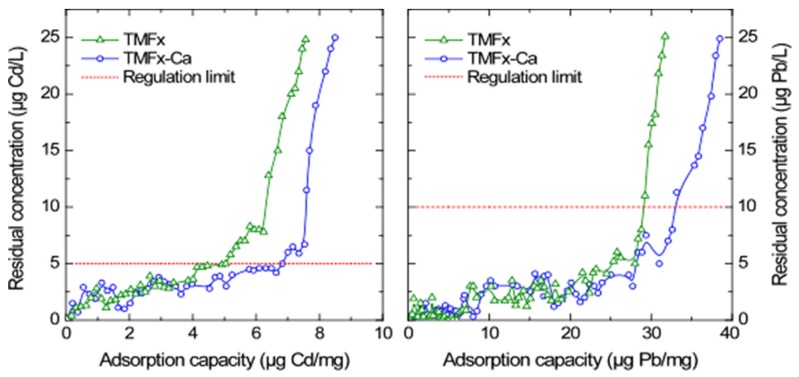
RSSCT breakthrough curves for the removal of Cd and Pb from water by adsorption, when using the NSF water matrix and for the pH value 7.2 ± 0.1.

The TMFx and TMFx-Ca adsorbents were subsequently tested in a continuous flow RSSCT setup that can simulate more accurately a full-scale adsorption process. According to the obtained breakthrough curves, as presented in Figure 7, the adsorption capacity of TMFx for the respective DWRL of Cd (i.e., the Q_5_ value) was 5.0 μg Cd/mg adsorbent, while for the TMFx-Ca material this value was increased to 6.8 μg Cd/mg adsorbent. Considering the slight variations in the pH values noted during the column experiments (i.e., 7.2 ± 0.1), the obtained efficiency is in good agreement with the aforementioned batch experiments. More specifically, the Q_5_ values of TMFx-Ca for the batch experiments at pH values 7 and 8 were 5.9 and 7.3 μg Cd/mg, respectively, whereas the corresponding value from the RSSCT experiment was 6.8 μg Cd/mg at pH 7.2.

The adsorption capacity of Pb using the TMFx material and for the corresponding DWRL (i.e., the Q_10_ value) was 28.5 μg Pb/mg, whereas this value was increased up to 35.0 μg Pb/mg for the case of TMFx-Ca adsorbent. However, in this case, the Q_10_ values of RSSCT experiments were found to be rather inconsistent to the respective from the batch tests; hence, an additional validation of the previous observation, regarding the (partial) precipitation of Pb at this pH value, can be suggested. Noting also that both examined adsorbents have presented much higher adsorption capacities for the case of Pb than for Cd, and considering moreover that both adsorption capacity and metal selectivity depend on the metal ion (M^n+^) radius, which is related to the M–O distance of the aqua complex [M(H_2_O)_y_^x+^] and to the hydration free energy (Table 4), the higher ion radius of Pb results in larger M–O distance and lower hydration free energy, which in turn favor its higher selectivity and adsorption capacity [3,28,29].

Based on these results, it is evident that the TMFx-Ca material exhibited higher adsorption capacity, due to the presence of more active sites, as verified by the higher negative surface charge density, since Cd and Pb are bound directly to these sites, as well as to the existence of an ion-exchange mechanism with the participation of more mobile Ca cation [12,13]. Furthermore, by comparing the XRD diagrams of the initial adsorbent materials with the saturated samples, i.e., after adsorption (presented as Figure S6), no significant structural variations were noticed, thus supporting the proposed mechanism.

The Q_DWRL_ value also determines the required “adsorbent cost” for the removal of Cd and Pb from water (Table 4). Since the current commercial cost of TMFx is around 8 €/kg and the corresponding cost of TMFx-Ca is estimated as 8.5 €/kg, due to the supplementary addition of CaCl_2_·2H_2_O, the cost of adsorbent required for the removal of 1 kg Pb from water by using the TMFx-Ca material can be calculated as:

### TMFx-Ca Consumption:

[1 kg Pb/ (35 μg Pb/mg TMFx-Ca)] = [1000 g Pb/ (35 g Pb/kg TMFx-Ca)] = 28.6 kg TMFx-Ca/kg Pb

Adsorbent cost = [28.6 kg TMFx-Ca/kg Pb] × [8.50 €/kg TMFx-Ca/kg Pb] = 243 €/kg Pb

More specifically, the cost of the adsorbent required for the removal of Pb (e.g., from natural water containing an initial concentration of 50 μg/L) at pH 7.2 ± 0.1 is calculated as:

Spent TMFx-Ca /10^3^ m^3^ = [10^3^ m^3^ × (50–10) mg Pb/ m^3^]/[35 × 10^3^ mg Pb/kg TMFx-Ca] = 1.15 kg

Adsorbent cost = (1.15 kg TMFx-Ca/10^3^ m^3^) × (8.50 €/kg TMFx-Ca) = 9.70 €/10^3^ m^3^

The energy and labor costs of the adsorption treatment process do not highly depend on the initial concentration of pollutants, but mostly from the quantity of treated water and from the energy and labor costs for each specific case study. For example, based on current Greek market prices, the respective energy and labor cost requirements were estimated to be approximately 30 ± 10 €/10^3^ m^3^ treated water. Therefore, from a techno-economic point of view, the removal of Cd and Pb from water when using the TMFx-Ca material is a feasible treatment process, considering the pH value and the initial concentrations commonly encountered in drinking water treatment, where these metals may exist in soluble forms and in low concentrations (in the order of several μg/L). To investigate the potential modification of water quality due to the presence of Ca and to the respective ion exchange process and the corresponding expected increase in pH value, these parameters were also determined in the effluent of TMFx-Ca column. The variation of Ca concentration, as well as the respective pH change, were considered rather negligible and within the standard deviation of experimental measurements, since the NSF water contains already rather high concentrations of Ca in order to simulate natural waters [30].

### 3.6. Leaching Tests

The saturated adsorbents after the end of RSSCT experiments were subsequently exposed to leaching experiments, following the typical procedure, as described in the EN 12457 protocol. The results shown in Table 5 indicate that, after use, both adsorbents could be further disposed as inert wastes, as their Cd and Pb contents are considered sufficiently stabilized. The recorded very low leachability values verify the rather high affinity of these metals, when removed by the TMFx or TMFx-Ca adsorbents and, furthermore, confirms that Pb removal can be attributed mainly to adsorption and not to its precipitation, because in the latter case this metal should present higher leachability values. In addition, the observed low values of leached calcium (<0.01 wt.%, Table 5) verify the active ion-exchange process, e.g., during cadmium and lead adsorption. These results were also confirmed by the fact that Ca^2+^ may present a higher affinity than Na^+^ for the surface of both sorbents, although this is still lower, when compared to other heavy metals, such as the examined Cd and Pb cases [31].

## 4. Conclusions

The results of this study demonstrate that the efficient adsorption of examined metals (Cd and Pb), leading to values under the respective DWRLs, as imposed by the legislation (i.e., the Q_DWRL_ values), is strongly related to:

✓ The negative surface charge density (mmol H^+^/g) of the applied adsorbent.
✓ The ionic radius of metals (M^n+^), which is related to the M–O distance of the relevant aqua complexes [M(H_2_O)_y_^x+^] and to the hydration free energy. A larger ionic radius (as in the case of Pb) results to greater M-O distance and to lower hydration free energy, which in turn can favor the improved selectivity and the higher Q_DWRL_-value.

The optimum pH range for the synthesis of these oxy-hydroxides is 9 ± 1, where the presence of excess OH^−^ can favor the deprotonation of oxy-hydroxide surfaces, thus maximizing the negative surface charge density. In addition, the synthesis of oxy-hydroxides in the presence of bivalent cations (i.e., Ca^2+^), instead of monovalent ones (i.e., Na^+^), may further increase the surface oxygen deprotonation and also, since the former cations are adsorbed, this can also result in the maintenance of active sites during the subsequent drying process.

More specifically, the synthesis of Ca-modified tetravalent manganese feroxyhyte in a solution containing around 1 g Ca^2+^/L (TMFx-Ca) was found to increase the negative surface charge density from 1.8 to 2.0 mmol H^+^/g. The influence of surface charge density modification on Cd and Pb uptake from a natural water matrix (NSF simulation) was also investigated by applying the Rapid Small-Scale Column Tests (RSSCTs), which are considered as more reliable for simulating full-scale treatment applications. The resulting adsorption capacities of TMFx material were Q_5_ = 5.0 μg Cd/mg adsorbent and Q_10_ = 28.5 μg Pb/mg, while the corresponding values for the TMFx-Ca adsorbent were Q_5_ = 6.8 μg Cd/mg and Q_10_ = 35.0 μg Pb/mg. Hence, the aforementioned higher negative charge of TMFx-Ca was proved to be sufficient enough to cause a notable increase of the respective adsorption capacities (i.e., 36% for the case of Cd and 23% for Pb). These capacities correspond to estimated adsorbent costs of 1250 €/kg Cd removed and 243 €/kg Pb removed, respectively. The TMFx and TMFx-Ca adsorbent materials appear suitable for drinking water treatment, as they do not modify significantly the quality characteristics of treated water. Additionally, both saturated (spent) adsorbents are considered as inert (stabilized) wastes, regarding their metals content, thus permitting easier final disposal.

## Figures and Tables

**Table 1:** Main physicochemical characteristics and the respective preliminary cost analysis of the prepared adsorbents.

Adsorbent	Chemical Composition				Surface Properties					Synthesis Cost
	Fe	Mn*	Ca	Na	IEP	PZC	[OH^−^]	[H^+^]	BET	
	(wt.%)						(mmol/g)		(m^2^/g)	€/kg
TMFx	38.8	12.5	0.1	3.2	5.9	7.6	1.0	1.8	301	8
TMFx-Ca	39.0	12.6	3.3	0.2	3.6	7.9	1.4	2.0	205	8.5

* Manganese valence: Mn(IV).

**Table 2:** Freundlich and Langmuir fitting parameters for the Cd adsorption isotherm data.

Adsorbent	pH	Q_5_ (μg Cd/mg)	Freundlich Parameters			Langmuir Parameters		
			K_F_ μg^.^mg^−1^(μg/L)^−1/n^	1/n	R^2^	K_L_ (L^·^μg^−1^)	Q_max_ (μg Cd/mg)	R^2^
TMFx	7	3.94	1.50	0.600	0.991	0.02	29.10	0.999
	8	5.91	2.47	0.542	0.993	0.05	26.76	0.993
TMFx-Ca	7	5.88	2.38	0.561	0.994	0.05	26.90	0.994
	8	7.29	3.34	0.485	0.990	0.09	23.50	0.991

**Table 3:** Freundlich and Langmuir fitting parameters, regarding Pb adsorption isotherm data.

Adsorbent	pH	Q_10_ (μg Pb/mg)	Freundlich Parameters			Langmuir Parameters		
			K_F_ μg·mg^−1^(μg/L)^−1/n^	1/n	R^2^	K_L_ (L·μg^−1^)	Q_max_ (μg Pb/mg)	R^2^
TMFx	7	158	39.65	0.599	0.966	0.037	625	0.941
	8	189	54.22	0.543	0.970	0.054	588	0.931
TMFx-Ca	7	184	50.17	0.565	0.963	0.052	588	0.929
	8	212	63.62	0.523	0.958	0.074	526	0.934

**Table 4:** Physicochemical characteristics of Cd and Pb related to the adsorption capacities of TMFx and TMFx-Ca materials and the respective adsorbent costs [28,29].

Metal	M^2+^ Ion Radius (Å)	M–O Distance of M(H_2_O)_6_^2+^ (Å)	Hydration Free Energy (kJ/mol)	Q_10_ (μg/mg*)		Adsorbent Cost (€/kg of Adsorbed/Removed Metal)	
				TMFx	TFMx-Ca	TMFx	TFMx-Ca
Cd	0.96	2.30	1979	5.0	6.8	1.600	1.250
Pb	1.20	2.54	1450	28.5	35.0	280	243

* Comparison of adsorption capacities taking into account equal breakthrough concentrations.

**Table 5:** Leaching characteristics of saturated TMFx and TMFx-Ca adsorbents after Cd and Pb sorption and removal from water, following RSSCT experiments.

Metal	Adsorbent	Load	Residual Ca	EN 12457-01		Threshold for Inert Wastes
		μg/mg	wt.%	pH	mg/kg	
Cd	TMFx	7.5	<0.01	7.2	0.02	0.04
	TMFx-Ca	8.5	0.015	7.2	0.03	
Pb	TMFx	31.7	<0.01	7.5	0.02	0.50
	TMFx-Ca	38.5	<0.01	7.6	0.01	

## Supplementary Materials

The following are available online at https://www.mdpi.com/1996-1944/13/7/1762/s1, Figure S1: Schematic representation of TMFx-Ca preparation using a 2-stage continuous flow reactor, Figure S2: ζ-potential curves of TMFx and TMFx-Ca materials, Figure S3: Determination of PZC by potentiometric mass titration curves [18], Figure S4: The surface charge distribution of TMFx material at various synthesis pH values, Figure S5: XRD diagram of the Pb precipitate and diffraction angles (triangles), according to ICDD/JCPDS database PDF#01-0687 [21], Figure S6: The XRD diagrams of saturated TMFx and TMFx-Ca sorbent materials (i.e., after adsorption). It is noted that the examined metals (i.e., Cd and Pb) have not provoked any significant variation in the spectra of saturated adsorbents, Text S1: Determination of Manganese Valence.
