# Supplementary material for: Improvement of Manganese Feroxyhyte’s Surface Charge with Exchangeable Ca Ions to Maximize Cd and Pb Uptake from Water
Source: Materials (Basel). 2020 Apr 9;13(7):1762. doi: 10.3390/ma13071762 (PMC7178661; doi:10.3390/ma13071762)
Supplement: Supplementary file 1 [file materials-13-01762-s001.pdf]

materials

MDPI

Supplementary Materials

Improvement of Manganese Feroxyhyte's Surface Charge with Exchangeable Ca Ions to Maximize Cd and Pb Uptake from Water

Evgenios Kokkinos [1], Chasan Chousein [2], Konstantinos Simeonidis [3], Sandra Coles [2], Anastasios Zouboulis [1] and Manassis Mitrakas [2,*]

1 Department of Chemistry, Aristotle University of Thessaloniki, 54124 Thessaloniki, Greece; evgenios@chem.auth.gr (E.K.); zoubouli@chem.auth.gr (A.Z.)
2 Department of Chemical Engineering, Aristotle University of Thessaloniki, 54124 Thessaloniki, Greece; cheng_auth_2013@hotmail.com (C.C.); sandycoles@hotmail.com (S.C.)
3 Ecoresources P.C., 15-17 Giannitson-Santaroza Str., 54627 Thessaloniki, Greece; simeonidis@ecoresources.gr
* Correspondence: manasis@eng.auth.gr

Received: 6 February 2020; Accepted: 7 April 2020; Published: date

Text S1: Determination of Manganese Valence

The valence of oxy-hydroxides Mn was chemically determined by the following procedure [1]: Depending on Mn content a sample of 200–400 mg were dissolved in 10 mL of 0.1 M Fe(II) solution and 3 mL of conc. H_2SO_4 under mild heating. Then, 10 mL of H_2O and 7 mL of conc. H_2SO_4 were also added. After cooling at room temperature, 10 mL of 85% H_3PO_4, 25 mL of H_2O and 3 drops of indicator (3 wt.% diphenylamine in ethanol) were added and the solution was titrated with the standard solution 0.05 N K_2CrO_7. The difference between this titration and the respective blank one was used for the Mn valence calculation, according to the following equations:

$$meq = (V_{blank} - V_{sample}) \times 0.05 \quad (1)$$

$$meq\ Mn^{3+} = m_{Mn}/MW_{Mn} = \%\ Mn \times m_{sample}/54.94 \quad (2)$$

$$meq\ Mn^{4+} = 2 \times (\%\ Mn \times m_{sample}/54.94) \quad (3)$$

$$Mn\ oxidation\ state = 3 + (meq - meq\ Mn^{3+})/\ (meq\ Mn^{4+} - meq\ Mn^{3+})$$

Ref.: Jiménez Mateos, J.M.; Macías, M.; Morales, J.; Tirado, J.L. Mn and Co substitution in δ-FeOOH and its decomposition products. *J. Mater. Sci.* **1990**, *25*, 5207–5214.

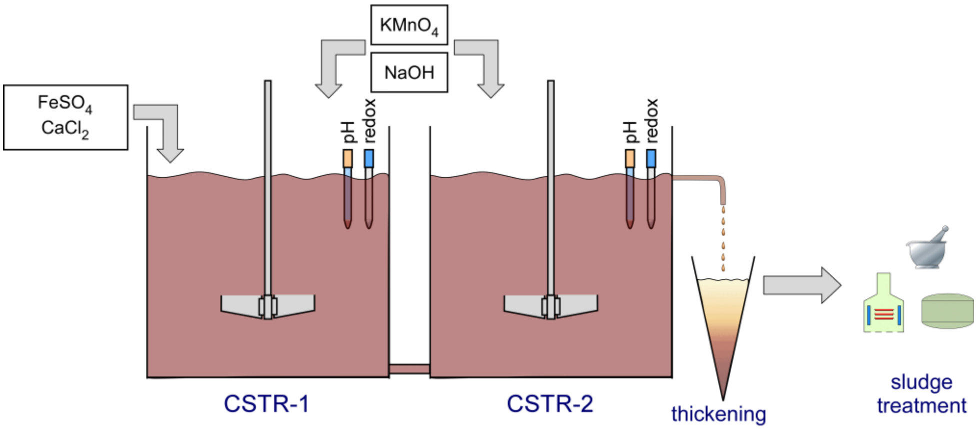

Figure S1. Schematic representation of TMFx-Ca preparation using a 2-stage continuous flow reactor.

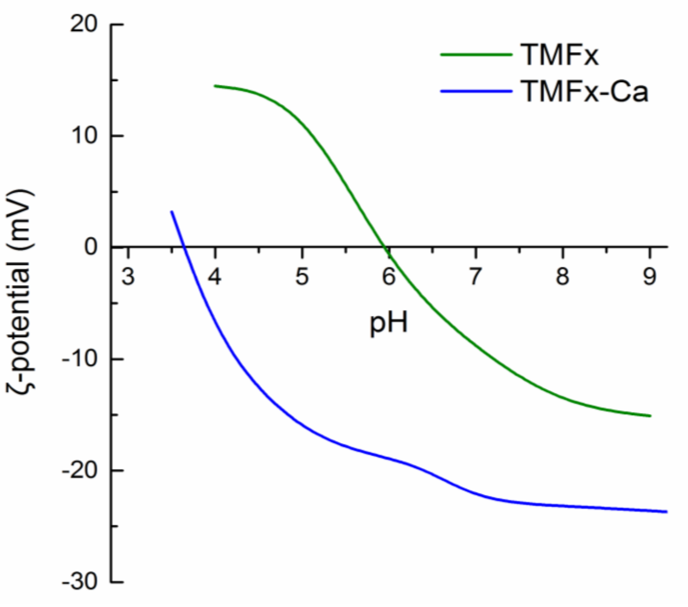

Figure S2. ζ-potential curves of TMFx and TMFx-Ca materials.

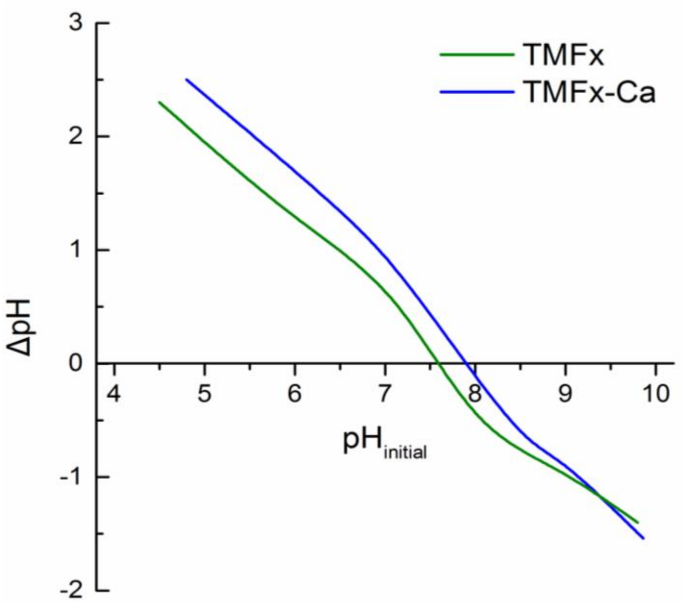

Figure S3. Determination of PZC by potentiometric mass titration curves [18].

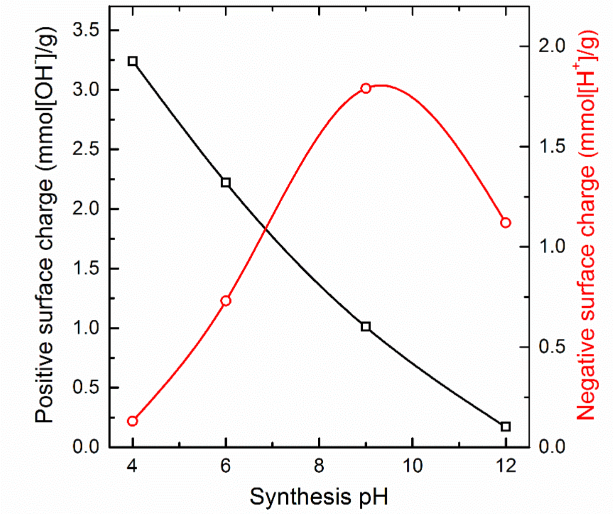

Figure S4. The surface charge distribution of TMFx material at various synthesis pH values.

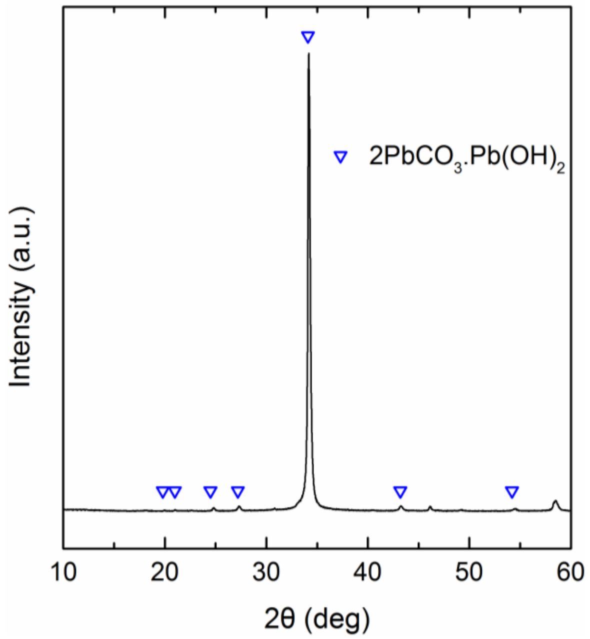

Figure S5. XRD diagram of the Pb precipitate and diffraction angles (triangles), according to ICDD/JCPDS database PDF#01-0687 [21].

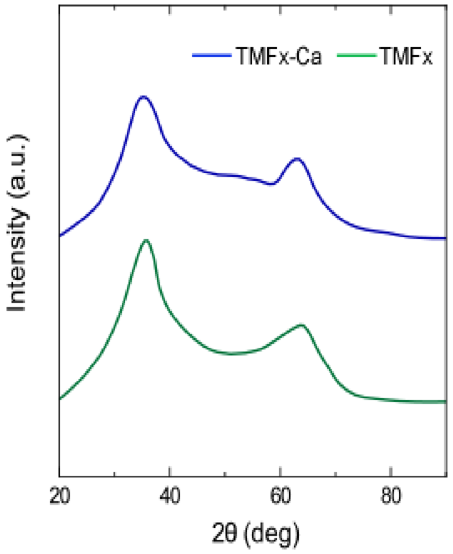

Figure S6. The XRD diagrams of saturated TMFx and TMFx-Ca sorbent materials (i.e., after adsorption). It is noted that the examined metals (i.e., Cd and Pb) have not provoked any significant variation in the spectra of saturated adsorbents.
